# Addressing the Unmet Needs of Patients With Rapidly Progressive Neurological Disease: A Case Report of Palliative Care in Creutzfeldt-Jakob Disease (CJD)

**DOI:** 10.7759/cureus.55228

**Published:** 2024-02-29

**Authors:** Justin R Price, Raya E Kheirbek

**Affiliations:** 1 Hospice and Palliative Care, University of Maryland School of Medicine, Baltimore , USA; 2 Medicine, University of Maryland School of Medicine, Baltimore , USA

**Keywords:** end-of-life and hospice care, symptom management, creutzfeldt-jakob disease, advance care planning, palliative care

## Abstract

Creutzfeldt-Jacob disease (CJD) is a rare neurodegenerative disorder that typically progresses rapidly and unrelentingly. Providing comfort and support for patients with CJD presents significant challenges for clinicians and caregivers. In comparison to the more typical disease progression experienced in dementias, the trajectory of CJD differs significantly. This case report delves into these differences and emphasizes the need for the development of guidelines for healthcare professionals and families who care for individuals with CJD. Such guidelines would help facilitate better care and support for patients and their families throughout the course of this devastating illness.

## Introduction

Creutzfeldt-Jakob disease (CJD) is a rare, fatal, neurodegenerative disorder caused by an accumulation of prions that leads to neuropathologically spongiform changes in the brain. In the United States, the incidence of CJD is roughly one to two cases per one million per year. The risk of CJD increases with age and people over the age of 55 are at higher risk [1]. The majority of cases of CJD (90%) occur sporadically while other cases are acquired or familial [2]. Sporadic CJD is a form of CJD that develops with no apparent reason. Iatrogenic CJD is acquired through surgical procedures or medical treatment such as contamination from brain surgery, corneal transplants, or grafts of dura mater [3].

This disease leads to progressive destruction of cortical neurons and is fatal. Early in the disease process, CJD patients can experience psychological symptoms in which they become withdrawn, apathetic, and have poor concentration. They can also display symptoms of depression, aggression, agitation, emotional lability, hallucinations, delusional behavior, personality change, insomnia, poor short-term memory, and anxiety [4]. As the disease progresses, patients develop symptoms of motor and cerebellar dysfunction [5]. These symptoms include dystonia, ataxia, chorea, primitive reflexes, rigidity, myoclonus, and parkinsonism [4]. CJD patients develop significant cognitive impairments that lead to rapid onset of progressive dementia [6]. Typical findings on EEG are periodic sharp discharge. The classic triad of progressive cognitive dysfunction, myoclonus, and periodic sharp wave complexes on EEG [7]. Further confirmation of the diagnosis would include elevated levels of 14-3-3 protein in the CSF. A neuropathological evaluation of brain tissue via autopsy is the definitive diagnosis [7].

## Case presentation

A 58-year-old White male software engineer was in a normal state of health until Thanksgiving Day 2022 when his significant other, who worked as a respiratory therapist, noticed a sudden change in his behavior. His significant other noted him taking T-shirts out of the closet and folding them obsessively. He told her he was not feeling well but did not elaborate. That evening he was able to cook dinner but did not eat anything. The following day his significant other noted he was disoriented with slurred and nonsensical speech. She thought he had a stroke and drove him to the emergency department. He was found to have elevated blood pressure which the clinicians believed was the cause of his symptoms. He was provided medications and recommended to follow up with his primary care physician.

Over the next several weeks, his cognitive status declined, he was more confused, and his dialogue became incoherent. His significant other noted he began to have trouble keeping his gait and was becoming rigid and unable to bend his knees. She also noted he would extend his arms and legs whenever startled. He was unable to walk, feed, dress, or bathe himself. She brought him back to the emergency department. What was initially thought to be delirium tremens due to alcohol withdrawal was refuted by the fact that he had not consumed alcohol for many weeks. CJD was suspected given the rapid nature of his clinical decline among many other diagnoses. The patient was transferred to the University of Maryland Medical Center for rapidly progressive dementia (RPD) with CJD as a potential cause. On arrival, he was minimally alert and was found to have an increased tone and generalized myoclonic movements. He was managed with lorazepam and admitted to the neurology unit.

**Figure 1:**
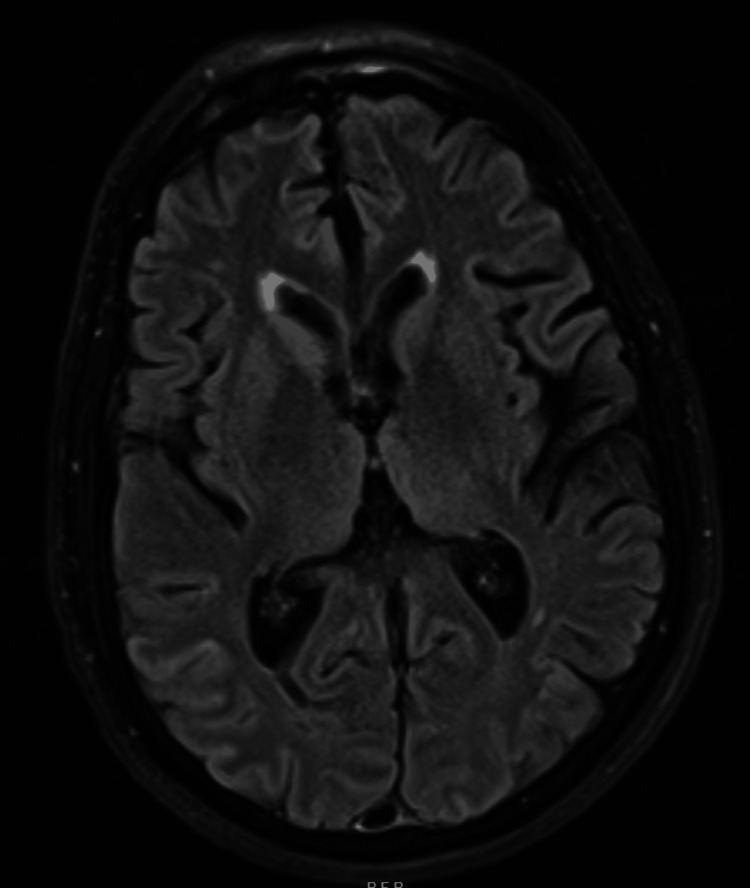
MRI T2 FLAIR showing hyperintensity in bilateral caudate nuclei, right putamen FLAIR: fluid-attenuated inversion recovery

**Figure 2:**
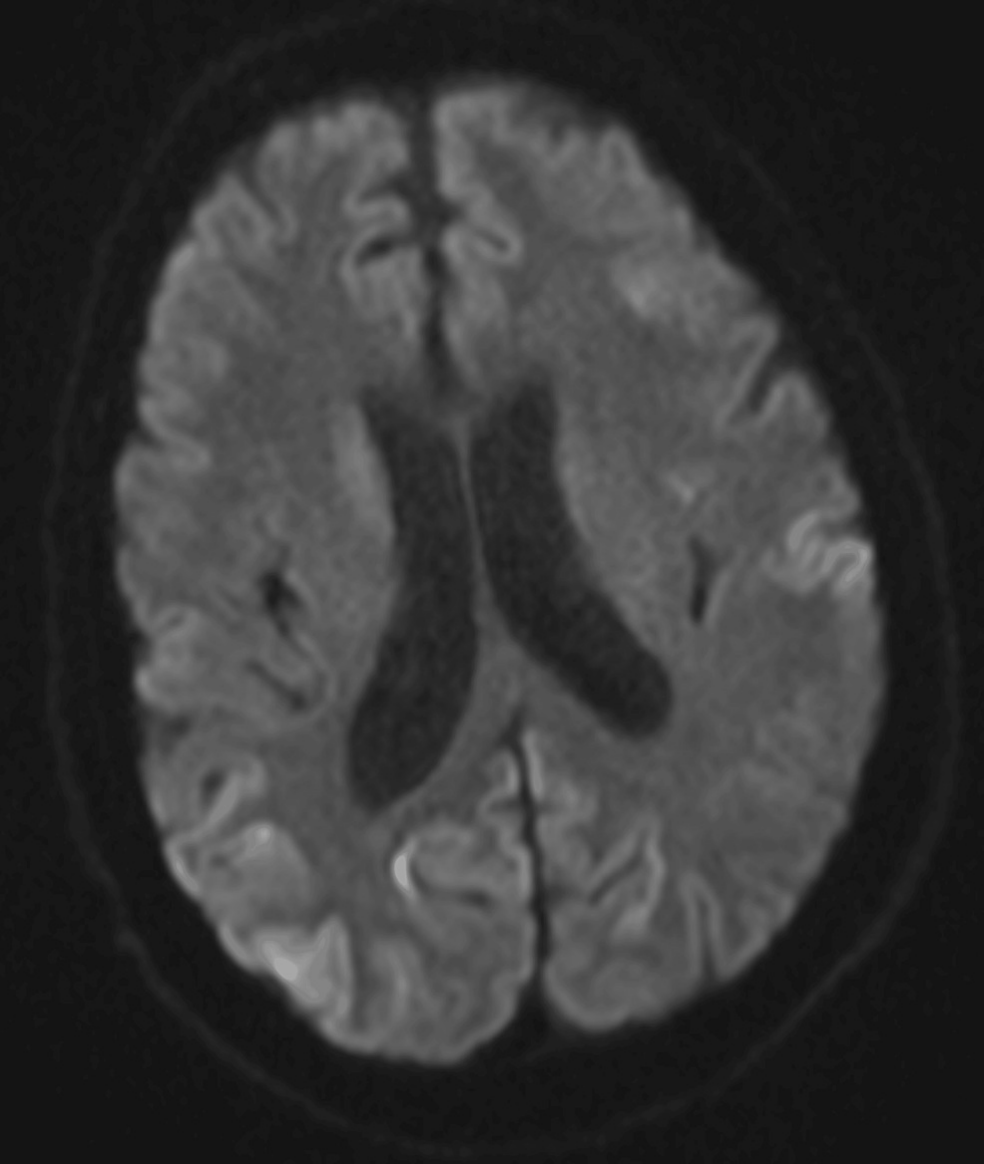
MRI showing diffusion restriction in bilateral hemispheres on DWI DWI: diffusion-weighted Imaging

**Figure 3:**
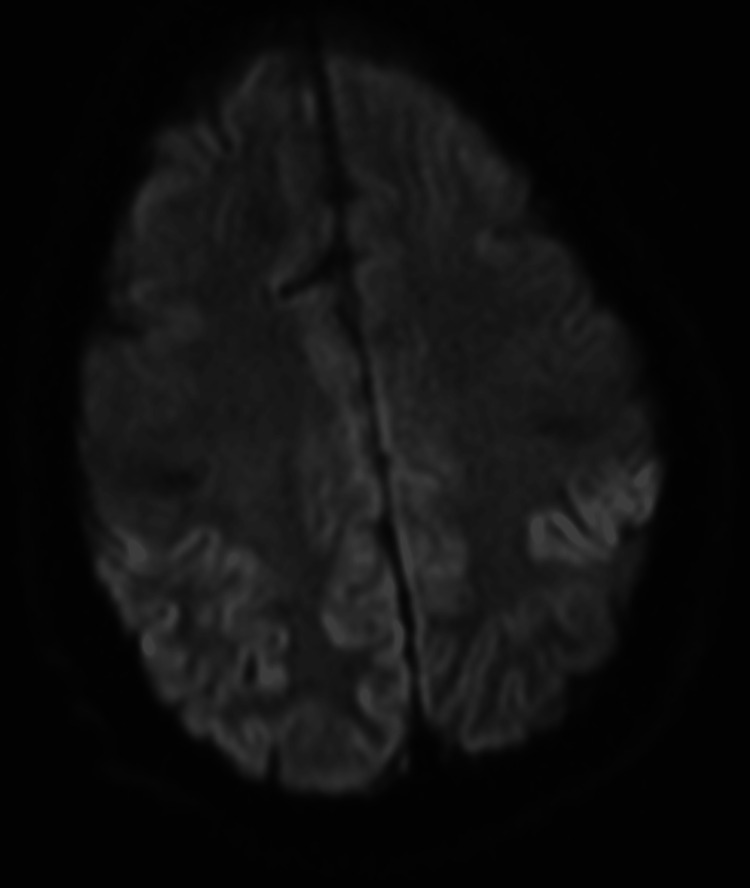
MRI showing diffusion restriction in bilateral hemispheres on DWI DWI: diffusion-weighted Imaging

The neurology team conducted a comprehensive work-up which included a lumbar puncture, MRI of the brain, and serological studies. The diagnosis of CJD was made based on the symptoms of rapid encephalopathy and extrapyramidal dysfunction. MRI of the brain showed an asymmetrical increase in T2 fluid-attenuated inversion recovery (FLAIR) signal intensities and restricted diffusion in the cortical ribbons of the bilateral cerebral hemispheres. This was more prominent in the anterior frontal and bilateral parietal lobes. There was additional restricted diffusion with hyperintensity on T2 FLAIR in the bilateral caudate nuclei, right putamen, and pulvinar and dorsomedial thalami (Figures 1-3). The findings were consistent with CJD.

**Figure 4:**
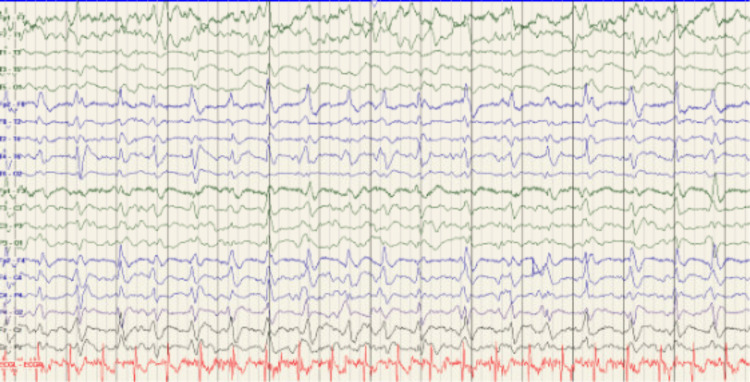
EEG Rhythmic or periodic pattern: Near continuous frontally predominant generalized periodic discharges (GPDs) are seen at 0.5-1.5 Hz for long duration, sharp and spiky, at times, asymmetric, with higher amplitude and clearer morphology on the right. Lateralized periodic discharges (LPDs) are seen on the right at 0.5-1.5 Hz for brief duration (less common over this 24-hour period)

The EEG showed lateralized periodic discharges at 0.5-1 HZ over the right hemisphere and generalized periodic discharges that were in the asymmetric and right-sided predominant (Figure 4).

Tables 1-2 illustrate CSF studies from the lumbar puncture. The results were negative for viral, rheumatological, or bacterial causes for the patient's clinical presentation. Lyme and *Cryptococcus* tests were negative (Table 3). Herpes simplex virus (HSV) type 1, HSV type 2, and Epstein-Barr virus (EBV) were negative (Table 4). There was evidence of T cell pleocytosis and CSF Tau protein was positive. T-tau protein in CSF was > 2,000 and the 14-3-3 gamma CSF was > 160,000 (Table 5). Arsenic, lead, and mercury were low (Table 6). A positive real-time quaking-induced conversion (CSF RT-QuIC) together with neuropsychiatric disorder met the definition for prion disease. Gag and corneal reflex were intact. The musculoskeletal exam was consistent with increased tone, and he was noted to have generalized myoclonic movements in the right lower extremity. Reflexes were decreased throughout.

**Table 1:** CSF Results 1 (01/04/23 14:26) H: high (elevated)

Tests	Value	Reference Range
RBC, CSF	15 (H)	0-0/mcL
WBC, CSF	14 (H)	0-5/mcL
CSF Comment	100 cells counted, Tube #4	_
Polymorphonuclear leukocytes % CSF	0	%
Lymphocytes % CSF	79	%
Monocytes % CSF	21	%
CSF Pre-centrifuge clarity	Clear	_
CSF Pre-centrifuge color	Slightly Xanthochromic	_
CSF Post-centrifuge clarity	Clear	_
CSF Post-centrifuge color	Slightly Xanthochromic	_

**Table 2:** Cerebrospinal fluid, Results 2 VDRL: Venereal Disease Research Laboratory; H: high (elevated) Note: There was a single band in the CSF and no bands in the serum, which indicates a negative result.

Test	Value	Reference Range
Glucose CSF	63	40-70 mg/dL
Protein CSF	88 (H)	12-60 mg/dL
VDRL CSF	Non-Reactive	Non-Reactive
RBC, CSF	6 (H)	0-0/mcL
WBC, CSF	18 (H)	0-5/mcL
CSF Comment	100 cells counted, tube #1	_
Polys % CSF	0	%
Lymphs % CSF	86	%
Monos % CSF	14	%
CSF pre-centrifuge clarity	Clear	_
CSF pre-centrifuge color	Slightly Xanthochromic	_
CSF post-centrifuge clarity	Clear	_
CSF post-centrifuge clarity	Slightly Xanthochromic	_
IgG, CSF	5.5	0.0-6.0 mg/dL
Albumin, CSF	31	3500-5200 mg/dL
Albumin index ratio	10.3 (H)	0.0-9.0 Ratio
IgG/Albumin ratio, CSF	0.18	0.09-0.25 Ratio
IgG Index	0.75 (H)	0.28-0.66
Oligoclonal bands, CSF	1 Negative	0-1 Bands
Interpretation, oligo bands	Negative (See Note)	_
IgG Synth Rate CSF	8.6 (H)	≤ 8.0
N-methyl-D-Aspartate Receptor Ab, CSF	< 1:1	< 1:1

**Table 3:** Urinalysis (01/01/23 14:40) ! indicates that it was converted to a Reflex Urine culture

Test	Results	Reference Range
Color	Yellow	Yellow
Appearance	Cloudy !	None
Specific gravity	1.017	1.003-1.035
pH	5.5	5.0-8.0
Glucose	Negative	Negative
Bilirubin	Negative	Negative
Urobilinogen	3.0 !	Negative
Ketones	Negative	Negative
Blood	3+ !	Negative
Protein	1+ !	Negative
Nitrite	Negative	Negative
Leukocyte esterase	Negative	Negative
WBC	3-5	0-5/hpf
RBC	>50 !	0-2/hpf
Squamous epithelial cells	0-2	0-2/hpf
Mucus	Large !	/hpf
Hyaline casts	6-10 !	/hpf
Amorphous crystals	Trace	/hpf
Bacteria	Negative	Negative

**Table 4:** CSF Results for herpes simplex virus and Epstein-Barr virus PCR: polymerase chain reaction

Test Name	Result
Herpes simplex virus 1 Subtype by PCR	Not Detected
Herpes simplex virus 2 Subtype by PCR	Not Detected
Epstein-Barr Virus by PCR	Not Detected

**Table 5:** CSF Test for Prion Disease RT-QuIC: Real-Time Quaking-Induced Conversion

Test Name	Result	Reference range for non-prion disease
RT-QuIC (CSF)	Positive	Negative
T-Tau protein (CSF)	>20000 pg/mL	0-1149 pg/mL
14-3-3 gamma (CSF)	>160000 AU/mL	<30-1999 AU/mL

**Table 6:** Blood tests (01/02/23 04:58) Ag/Ab: antigen/antibody

Tests	Results	Reference Range
Arsenic	<10.0	≤ 12.0 ug/L
Lead	<2.0	≤ 4.9 ug/dL
Mercury	3.6	≤ 10 ug/L
HIV Ag/Ab	Non-reactive	Negative

**Table 7:** Blood tests (01/04/23 14:19)

Tests	Results
Amphiphysin antibody, Serum	Negative
CV2.1 antibody IgG	<1:10
Paraneoplastic syndrome antibody PNL with reflex	None detected
SOX1 antibody IgG	Negative

**Table 8:** Blood tests carried out day-wise RT-PCR: reverse transcriptase-polymerase chain reaction

	01/1/23	01/2/23	01/3/23	01/4/23
Blood Culture Organism ID Panel	*Staphylococcus* species contaminate	Not done	Not done	Not done
*Cryptococcus* AG CSF or Serum	Not done	Not done	Not done	Negative
Lyme DNA PCR	Not done	Not done	Not done	Not detected
*Streptococcus* Group A PCR	Not detected	Not done	Not done	Not done
*Treponema pallidum* IgG antibody	Not done	Non-Reactive	Not done	Not done
Varicella Zoster RT PCR	Not done	Not done	Not done	Not detected
CSF Culture (Gram Stain)	Not done	Not done	Not done	No growth

**Table 9:** Blood chemistry (01/01/23 15:46) BUN: blood urea nitrogen; eGFR: estimated glomerular filtration rate; AST: aspartate aminotransferase; ALT: alanine transaminase; H: high (elevated); L: low

Tests	Values	Reference Range
Sodium	140	136-145 mmol/L
Potassium	3.4 (L)	3.5-5.1 mmol/L
Chloride	108 (H)	98-107 mmol/L
CO2 Total	30	21-30 mmol/L
Anion Gap	3 (L)	4-16
BUN	18	9-20 mg/dl
Creatinine	0.97	0.66-1.25 mg/dL
eGFR	91	≥60
Glucose, Bld	124 (H)	70-99 mg/dL
Calcium	9.3	8.6-10.2 mg/dL
Magnesium	1.9	1.6-2.6 mg/dL
Phosphorus	3.7	2.5-4.5 mg/dL
Total Protein	6.1 (L)	6.3-8.2 g/dL
Albumin	3.3 (L)	3.5-5.2 g/dL
AST	40	17-59 units/L
ALT	35	0-49 units/L
Bilirubin, total	1.2	0.3-1,2 mg/dL
Bilirubin, direct	0.6 (H)	≤ 0.4 mg/dL
Alkaline phosphatase	77	38-126 units/L
Lactate	1.4	0.5-2.2 mmol/L

**Table 10:** Comprehensive metabolic panel H: high

Test	01/02/23 04:58	01/03/23 04:36
Hemoglobin A1C	Not done	5.7 (H)
Thyroid-stimulating hormone	0.62	Not done
Thyroid peroxidase antibody	0.4	Not done

**Table 11:** Inpatient medications SBP: systolic blood pressure; SC: subcutaneous; NG: nasogastric tube; PRN: pro re nata (not scheduled)

Drug Name	Dose	Form	Instructions
Amlodipine	5 mg	Orally	Per NG tube daily
Labetolol	10 mg	IV	Once; Hold if SBP< 100 mm HG or HR < 50
Lacosamide (Vimpat)	400 mg	IV	IV pushed at a rate up to 80 mg/min; IV push 100 mg over two minutes; IV push 200 mg over three minutes; IV push 400 mg over five minutes
Levetiracetam (Keppra)	2000 mg	IV	In sodium chloride 0.9 % 100 mL; Do not refrigerate.
Lidocaine (Xylocaine)	2% injection 10 ML	SC	Please place full vial at bedside for LP
Lorazepam (Ativan)	1 mg	IV	If given IV push, maximum rate 2 mg/min.
Potassium Chloride	10 MEQ	IV	Every two hours; Route: IV (Start: 01/01/23 1845 End: 01/02/23 0344)
Thiamine (Vitamin B-1)	200 mg	IV	Every eight hours; Route: IV (Start: 01/02/23 0045 End: 01/03/23 1628)
Valproate (Depakene) solution	1000 mg	Orally	Per NG tube, take with food or water. Do not mix syrup with carbonated beverages.
Hydralazine	25 mg	Orally	Per NG tube three times daily
Haloperidol Acetate (Haldol)	0.5 mg	IV	0.5 mg every six hours PRN
GuaiFENesin-dextromethorphan (Robitussin DM)	100-10 mg/5mL syrup, 10 mL dose	Orally	Per NG tube every four hours PRN
Clonidine (Catapres)	Tablet 0.1 mg	Orally	Per NG tube three times daily
Chlorhexidine gluconate (Peridex)	0.12 % oral rinse, 15 mL dose	Orally	15 mL two times daily
Atropine 1% ophthalmic solution	2 drop dose	Orally	Two drops every four hours PRN; Route: SL
Acetaminophen (Tylenol)	650 mg dose	Orally	Per NG tube every four hours PRN
Albuterol 2.5 mg/3mL nebulizer solution	2.5 mg dose	Inhaled	Every six hours PRN

**Table 12:** Hematology

Laboratory Test	01/01/23	01/04/23	Reference Range
WBC	9.3	10.4	4.5-11 K/mcL
Hemoglobin	13.0	14.0	12.6-17.4 g/dL
Hemoglobin (Hematocrit)	38.7	41.6	37-50 %
Platelets	160	183	153-367 K/mcL
Neutrophil Absolute	7.40	7.70	1.70-7.30 K/mcL
Neutrophil Percentage	79.6	74.5	42.6-74.5%
Lymphocytes Absolute	1.0	1.3	1.3-3.5 K/mcL
Lymphocyte Percentage	10.2	12.8	20.8-50.5%
Monocyte Absolute	0.7	1.0	0.1-0.7 K/mcL
Monocyte Percentage	7.9	9.3	2.0-10.3%
Absolute Basophil Count	0.0	0.0	0.0-0.1 K/mcL
Basophil Percentage	0.3	0.4	0.2-1.0%
Eosinophil Absolute	0.2	0.2	0.0-0.2 K/mcL
Eosinophil Percentage	1.6	2.3	0.9-2.9%
Mean Corpuscular Hemoglobin (MCH)	31.2	31.3	28-33 pg
Mean Corpuscular Hemoglobin Concentration (MCHC)	33.6	33.7	33-36 g/dL
Mean Corpuscular Volume (MCV)	92.8	93.1	80-96 fL
Mean Platelet Volume (MPV)	10.5	10.4	9.4-12.4 fL
Red Blood Cell (RBC)	4.17	4.47	4.00-5.70 M/mcL
Red Cell Distribution (RDW)	12.5	12.5	12-15.2%
Absolute Immature Granulocytes	0.00	0.10	0.00-0.03 K/mcL
Immature Granulocyte Percentage	0.4	0.7	0.0-0.5%

Palliative care

The palliative care team was consulted by the neurology team to clarify the goals of care, establish a plan of care that aligned with the patient’s goals, values, and preferences, and provide recommendations for symptom management.

Goals of Care

Due to persistent delirium and cognitive impairment, he was unable to engage in a meaningful goal-of-care discussion. The palliative care specialist followed the SPIKES (setting, perception, invitation or information, knowledge, empathy, and summarize or strategize) steps: (i) Setting up the interview; (ii) assessing the patient’s perception, (iii) obtaining the patient’s Invitation, (iv) giving knowledge and information to the patient, (v) addressing the patient’s emotions with empathetic responses, (vi) strategy and summary

The patient’s significant other stated that he was an engineer. She described him as outgoing, having many friends, enjoying the outdoors and hiking. She expressed frustration over the challenges that occurred in identifying the diagnosis and the lack of treatment options. Admittingly, at first, she was in disbelief of the diagnosis of CJD but then accepted the diagnosis.

The palliative care specialist explained that the patient's condition was fatal, and his mental status would continue to decline, which put him at high risk for aspiration and respiratory distress. Respiratory distress could lead to artificial life support requiring mechanical intubation and given his progressive decline in mental status, he would be dependent on artificial life support for the rest of his life. He could not safely swallow due to his progressively declining mental status, and he would need artificial nutrition via a PEG tube. The significant other said that there was no advance directive but within the last few years, they discussed their goals of care together and dependence on artificial life support was not an acceptable quality of life for the patient. The significant other expressed that the patient valued his mind and his independence the most. Quality of life to the patient meant being able to interact with his friends and family. She said he would not want to be dependent on artificial life support like mechanical ventilation or artificial nutrition via a percutaneous endoscopic gastrostomy (PEG) tube as these interventions did not align with his values. The palliative care specialist discussed the hospice philosophy of care, which is a plan of care focused on prioritizing symptom management and comfort. The significant other agreed that this plan of care aligned with his values, goals, and preferences at this time. It was agreed upon to change the patient code status to do not resuscitate, do not intubate, and palliative and supportive symptoms only. Additional changes to the plan of care from the discussion with the palliative care specialist included no additional procedures including imaging, lab draws, EEG monitoring, or medications that did not align with providing comfort. Plan of care focused on ensuring patient quality of life, reducing suffering, and symptom management.

The significant other expressed that the patient would have wanted to be at home around friends and family for end-of-life care. The palliative care specialist discussed the different levels of care with hospice. It was discussed that the hospice health care team, which comprised a physician, nurse, social worker, and chaplain, could assist in meeting the goals of care. The hospice healthcare team could provide symptom management as well as equipment, supplies, and spiritual and bereavement support. It was clear that home hospice aligned with the patient's goals of care and the palliative care team worked in coordination with the palliative care social worker and hospice agency.

Symptom Management

Unlike other neurodegenerative diseases like Alzheimer’s disease, Parkinson’s disease, or Lewy body disease, CJD symptoms progress rapidly. A palliative care team can provide guidance in symptom management. Non-pharmacological management of CJD involves a collaborative approach from an interdisciplinary team. Social workers assist in completing advance directives and obtaining disability and other benefits. Chaplaincy is beneficial for anticipatory grief and spiritual needs. Reflective listening and counseling can help caregivers cope with the emotional and psychological aspects of caring for someone with CJD. This can involve having a supportive professional or counselor who can provide guidance and assistance in managing the stress and emotional toll associated with the disease. Music therapy can be a valuable tool in creating a more therapeutic care environment for individuals with CJD. Music has been known to have a positive impact on mood, emotions, and overall well-being. It can help promote relaxation, reduce anxiety, and improve the quality of life for both the individual with CJD and their caregiver. Aromatherapy involves the use of essential oils and scents to promote relaxation, reduce stress, and improve emotional well-being. Certain essential oils such as lavender or chamomile may have calming effects and can be used in a caregiving setting to create a more soothing environment. Establishing consistent daily routines can help individuals with CJD feel more secure and may reduce behavioral manifestations associated with the disease. Providing a predictable and structured environment can help individuals with CJD maintain a sense of stability and familiarity.

The patient had symptoms of delirium, restlessness, and agitation at times. He had significant secretions and required frequent suctioning. His non-verbal symptoms of pain and anxiety were facial grimacing, restlessness, hyperventilation, and agitation. He would exhibit signs of myoclonic seizures in his right lower extremity. The pharmacologic management of symptoms associated with CJD lacked specific guidelines and evidence. The patient needed regular reassessment of the risks, benefits, and response to pharmacotherapies to make necessary adjustments. For delirium and agitation, typical antipsychotics such as haloperidol 0.5-1 mg were provided orally and intravenously. There were times that lorazepam 1-2 mg IV was also provided for uncontrolled agitation. Secretions were managed with scopolamine patch and myoclonus was managed with antiepileptics like levetiracetam or valproate orally and intravenously. Clonazepam was considered as an alternative but not used. These medications were initiated at the lowest effective dose, and the patient’s response was monitored closely. The approaches outlined above reflect general guidance as there are no disease-specific guidelines.

The palliative care team discussed the hospice option. The significant other believed he would want to be at home. The team provided anticipatory guidance about the potential challenges of managing his symptoms at home including frequent suctioning and close management of his delirium, agitation, and myoclonic seizures. While the complexity of his symptom management would have been best managed in an inpatient hospice unit or a nursing home that had around-the-clock nursing care, his significant other and her daughter, who worked as a critical nurse, both were comfortable meeting his level of care at home. The palliative specialist reviewed each medication and provided guidance on when to give each medication and what side effects to be aware of. For example, it was recommended if the patient displayed agitation or restlessness, they were to start haloperidol 1-2 mg orally prn (pro re nata) and consider starting lorazepam 1-2 mg orally prn if haloperidol was ineffective. Haloperidol and lorazepam have a sublingual form which could be provided if there were challenges in swallowing. It was discussed that for seizure and/or myoclonus, they could start lorazepam 1-2 mg orally prn and increase as needed. In addition, midazolam could be considered as it could be provided intranasally. It was advised to contact the hospice agency if there were any additional questions or concerns. It was also shared that if his significant other was unable to manage his symptoms at home, then transfer to an inpatient hospice unit was an alternative. The palliative social worker met with the significant other to discuss home hospice and the equipment and supplies that would be needed. The medications recommended for hospice care are given in Table 13.

**Table 13:** Medications recommended for hospice care prn: pro re nata; SL: sublingual; SSRI: selective serotonin reuptake inhibitor

Symptom Type	Treatment	Starting Doses/ Route	Comments
Delirium	Non-pharmacological: Reorientation, hydration, mobilization, sleep strategies Pharmacological: 1^st^ line Haloperidol, Lorazepam, Olanzapine, Risperidone, Quetiapine	1-2 mg orally prn, 1-2 mg orally prn 1.25 -2.5 mg orally, 0.5-1 mg orally 25 mg orally daily; Haloperidol and Lorazepam can be provided SL (Intensol)	Can exhibit hyperactive, mixed, or hypoactive delirium
Seizures	Benzodiazepines, Lorazepam, Diazepam, Clonazepam, Midazolam	1-2 mg orally prn, 5 mg orally prn provided intranasally	Treat Myoclonus
Depression	Early referral to psychiatry, social support; SSRI first line if life expectancy is > 2-3 weeks Escitalopram, Citalopram, Sertraline, Mirtazapine	5 mg orally 10 mg orally, 25 mg orally, 7.5 mg orally	SSRI cause dry mouth, GI disturbances Avoid Wellbutrin, lower seizure threshold
Breakthrough Pain	Opioids: Morphine, Hydromorphone, Oxycodone, Methadone	5 mg orally prn, 1 mg orally prn, 2.5 mg orally prn, 1-10 mg orally prn All opioids listed above can be given SL (Intensol)	Monitor for constipation. worsening delirium, respiratory, depression

Home Hospice Journey

The hospice agency provided support with equipment, supplies, and medications and further instructions on how to give the medications. The patient's most common symptoms at home were restlessness, agitation, and seizures. His significant other kept meticulous records of what she gave the patient. The significant other reported that she gave lorazepam, haloperidol, and morphine and continued the scopolamine patch. For home hospice, the palliative care specialist recommended intranasal midazolam for breakthrough seizures and provided education for the significant other on when to administer midazolam. In the last week of his life, he was weaker, less agitated, and less responsive and did not require as much symptom management. The patient died two weeks after discharge from the hospital. The significant other said he died peacefully at home surrounded by his family. She said she had joined a social media support group that included families who had loved ones with CJD. She had also utilized The CJD Foundation as a source for additional information about CJD. An autopsy was performed that confirmed the diagnosis of sporadic CJD.

## Discussion

CJD is a rare neurodegenerative disorder caused by the accumulation of an altered prion protein. The disease leads to a destruction of cortical neurons and is fatal. CJD should be considered in any patient with acute onset of prominent motor and or cerebellar dysfunction [5]. Most neurodegenerative dementias develop slowly which warrants an unhurried outpatient evaluation process. The most common slowly progressive dementia is Alzheimer’s disease and other examples of slow progressive dementia are Parkinson’s disease, frontotemporal dementia, and subcortical ischemia vascular disease, which typically progress over years. Unlike these dementias, CJD's common feature is a rapid neuropsychiatric decline with death usually occurring within one year of symptom onset [8]. Patients with CJD most commonly present with cognitive dysfunction followed by cerebellar, behavioral, constitutional, sensory, motor, and visual dysfunction [5]. Palliative care specialists can help manage these rapidly developing complications [9]. 

Palliation is the only treatment and early diagnosis is important in referring patients to palliative and end-of-life services. Some reasons for late referral to a palliative care specialist could be the rapid rate of clinical decline and the sense of impotence around the lack of treatment options. Management of physical and psychosocial symptoms and family bereavement is complex and challenging. Palliative care consultation at CJD diagnosis can provide advanced care planning, anticipatory guidance, and symptom management [10]. Palliative care provides a holistic approach to patient care, as it addresses a patient’s physical, psychological, social, spiritual, and cultural aspects of care [11]. Our palliative care team consists of physicians, nurse practitioners, nurses, pharmacists, social workers, and chaplains. The palliative care team not only addresses patients’ goals of care and provides advance care planning for patients with advanced and serious illnesses, but also supports those that are imminently dying through expertise in symptom management and social support with legacy activities and coordination with hospice agencies [12]. Palliative care social workers can help expedite advance directive completion and pursuit of disability and other benefits. Chaplain support helps with anticipatory grief and spiritual support. The palliative care team provides reflective listening and/or psychologic counseling and can help caregivers cope with the rapid personality and functional changes associated with CJD. The palliative care team will also recommend integrative medicine which includes music therapy and aromatherapy. Many studies reveal that palliative care improves the quality of life and symptom burden for patients with advanced disease [13].

Patients and their families can benefit from palliative care services, but palliative care specialists face the challenge of having limited knowledge about CJD. Therefore, palliative care interventions will need to be refined with an interdisciplinary team of specialists which includes psychiatry, neurology, internal medicine, speech, and physical therapy. This comprehensive approach can ensure that the needs of patients and their families are met but requires further testing for utility and effectiveness [14]. 

Early in the disease process, patients with CJD can have psychiatric symptoms and become withdrawn, apathetic, and have poor concentration. They can also have symptoms of depression, aggression, agitation, emotional liability, hallucinations, delusional behavior, personality change, insomnia, poor short-term memory, and anxiety [4]. Therefore, referral to psychiatric services in the early stage of illness and utilizing mental health services can help in managing complex psychological issues [4]. 

There are no specific guidelines nor evidence that specific pharmacologic and non-pharmacologic management of CJD-related symptoms is effective [10]. Palliative care specialists can assist in providing recommendations for non-pharmacological approaches to symptom management of CJD. This requires an individualized approach based on the patient’s needs and goals of care. This can include referral to physical therapy, speech therapy, occupational therapy, or music therapy. Patients with CJD will often exhibit hyperactive and mixed delirium and non-pharmacological methods can help. This includes reorientation, hydration, mobilization, and sleep strategies [15]. First-line pharmacological therapy includes haloperidol. Typical starting doses are 1-2 mg as needed for agitation. Scheduled haloperidol 2 mg every six or 12 hours may be added for select patients. Consideration should be given to lorazepam 1-2 mg as needed for severe cases of agitation. Atypical antipsychotics like olanzapine starting dose of 2.5 mg daily, risperidone starting dose of 1 mg nightly, and quetiapine starting dose of 25 mg daily can also be considered [10]. 

Seizures are an uncommon finding in sporadic CJD, occurring in less than 15% [5]. For myoclonus and seizures, a palliative care specialist will consider the impact of side effects of anti-epileptic drugs, pill burden, impending loss of oral intake, and potential benefits [16]. Benzodiazepines are the most commonly used anti-epileptic drugs at end-of-life. Benzodiazepines can be dosed within five minutes of a seizure, after which it can be scheduled or provided as needed depending on the clinical setting. CJD patients lose their ability to take medications orally and so there are other ways that benzodiazepines can be provided. For example, diazepam can be given rectally or intranasally. Midazolam can be given intranasally [17]. Clonazepam can be given as an ODT and Lorazepam comes as a concentrate and can be given SL. End-of-life seizures can be traumatic for the family and family education by the palliative care specialist can help with management.

## Conclusions

Providing interdisciplinary palliative care for patients with CJD is essential. The family in the current case expressed gratitude for the palliative care specialist being a source of support and providing guidance as his clinical status declined and symptoms worsened. The family also valued the presence and availability of the palliative care team as they did not feel abandoned. Palliative care helps in providing education on disease trajectory, anticipating and managing symptoms, aligning care goals with patient values and preferences, and supporting loved ones in anticipatory grief and bereavement after death. While more research is needed to determine how palliative care can be best effective for patients with CJD, the importance of this care cannot be overstated.

## References

[1] (2023). Creutzfeldt-Jakob Disease, Classic (CJD). https://www.cdc.gov/prions/cjd/index.html.

[2] Eimer J, Vesterbacka J, Savitcheva I, Press R, Roshanisefat H, Nowak P (2018). Nonopportunistic infection leading to rapidly progressive dementia in a patient with HIV/AIDS: a case report. Medicine (Baltimore).

[3] Wieser HG, Schindler K, Zumsteg D (2006). EEG in Creutzfeldt-Jakob disease. Clin Neurophysiol.

[4] de Vries K, Sque M, Bryan K, Abu-Saad H (2003). Variant Creutzfeldt-Jakob disease: need for mental health and palliative care team collaboration. Int J Palliat Nurs.

[5] Geschwind MD, Haman A, Miller BL (2007). Rapidly progressive dementia. Neurol Clin.

[6] Nagata T, Shinagawa S, Kobayashi N, Kondo K, Shigeta M (2022). A case of V180I genetic mutation Creutzfeldt Jakob disease (CJD) with delusional misidentification as an initial symptom. Prion.

[7] Iwasaki Y (2017). Creutzfeldt-Jakob disease. Neuropathology.

[8] Haywood AM (1997). Transmissible spongiform encephalopathies. N Engl J Med.

[9] De Vries K, Cousins E, Harrison Dening K (2021). Palliative care in Creutzfeldt-Jakob disease: looking back, thinking ahead. BMJ Support Palliat Care.

[10] McQuain JA, Galicia-Castillo MC, Morris DA (2020). Palliative care issues in Creutzfeldt: Jakob disease #389. J Palliat Med.

[11] (2024). Clinical Practice Guidelines for Quality Palliative Care. Clinical Practice Guidelines for Quality Palliative Care, 4e.

[12] (2023). What is Palliative Care?. https://getpalliativecare.org/whatis/.

[13] Kavalieratos D, Corbelli J, Zhang D (2016). Association between palliative care and patient and caregiver outcomes: a systematic review and meta-analysis. JAMA.

[14] Harrison KL, Garrett SB, Gilissen J, Terranova MJ, Bernstein Sideman A, Ritchie CS, Geschwind MD (2022). Developing neuropalliative care for sporadic Creutzfeldt-Jakob disease. Prion.

[15] Hui D, Agar M, Maeda I (2023). Should neuroleptics be used in patients with delirium seen by palliative care?. J Pain Symptom Manage.

[16] Sharma A, Besbris JM, Kramer NM, Daly FN, Singhal D, Jones CA, Mehta AK (2021). Top ten tips palliative care clinicians should know about seizures at the end of life. J Palliat Med.

[17] Kälviäinen R, Reinikainen M (2019). Management of prolonged epileptic seizures and status epilepticus in palliative care patients. Epilepsy Behav.

